# Seizure Prediction in Genetic Rat Models of Absence Epilepsy: Improved Performance through Multiple-Site Cortico-Thalamic Recordings Combined with Machine Learning

**DOI:** 10.1523/ENEURO.0160-21.2021

**Published:** 2022-02-08

**Authors:** Björn Budde, Vladimir Maksimenko, Kelvin Sarink, Thomas Seidenbecher, Gilles van Luijtelaar, Tim Hahn, Hans-Christian Pape, Annika Lüttjohann

**Affiliations:** 1 Institute of Physiology I, University of Münster, 48149 Münster, Germany; 2 Neuroscience and Cognitive Technology Lab, Innopolis University, 42055 Innopolis, Republic of Tatarstan, Russia; 3 Institute for Translational Psychiatry, University of Münster, 48149 Münster, Germany; 4 Donders Institute for Brain, Cognition and Behavoiur, Radboud University Nijmegen, 6525 GD Nijmegen, The Netherlands

**Keywords:** absence epilepsy, artificial neuronal network, GAERS, random forest, somatosensory cortex, spike and wave discharges

## Abstract

Seizure prediction is the grand challenge of epileptology. However, effort was devoted to prediction of focal seizures, while generalized seizures were regarded as stochastic events. Long-lasting local field potential (LFP) recordings containing several hundred generalized spike and wave discharges (SWDs), acquired at eight locations in the cortico-thalamic system of absence epileptic rats, were iteratively analyzed in all possible combinations of either two or three recording sites, by a wavelet-based algorithm, calculating the product of the wavelet-energy signaling increases in synchronicity. Sensitivity and false alarm rate of prediction were compared between various combinations, and wavelet spectra of true and false positive predictions were fed to a random forest machine learning algorithm to further differentiate between them. Wavelet analysis of intracortical and cortico-thalamic LFP traces showed a significantly smaller number of false alarms compared with intrathalamic combinations, while predictions based on recordings in Layers IV, V, and VI of the somatosensory-cortex significantly outreached all other combinations in terms of prediction sensitivity. In 24-h out-of-sample recordings of nine Genetic Absence Epilepsy Rats from Strasbourg (GAERS), containing diurnal fluctuations of SWD occurrence, classification of true and false positives by the trained random forest further reduced the false alarm rate by 71%, although at some trade-off between false alarms and sensitivity of prediction, as reflected in relatively low F1 score values. Results provide support for the cortical-focus theory of absence epilepsy and allow the conclusion that SWDs are predictable to some degree. The latter paves the way for the development of closed-loop SWD prediction-prevention systems. Suggestions for a possible translation to human data are outlined.

## Significance Statement

Seizure prediction was declared the grand challenge of epileptology. While much effort was devoted to the prediction of focal seizures, generalized seizures were regarded as stochastic events. Results of this study demonstrate that above chance prediction of generalized spike and wave discharges (SWDs) is possible in long-lasting, pseudoprospective 24-h recordings of absence epileptic rats, by means of wavelet analysis of local field potential (LFP) traces acquired near the proposed cortical initiation network in S1 and further classification of true and false positive detections by a trained random forest machine learning algorithm. Moreover, as lower SWD prediction performance was achieved by analysis of LFP traces distant to S1, the study provides evidence supporting the cortical focus theory of absence epilepsy.

## Introduction

Epilepsy is a neurologic disorder characterized by infrequent, short-lasting periods of either local or generalized, hypersynchronous brain activity which can be recorded in the electroencephalogram. Depending on the type and nature of these seizures they either go along with a loss of behavioral control in the form of tonic or clonic convulsions and/or with a loss of consciousness. As a majority of patients diagnosed with epilepsy report the uncertainty of when a seizure attack will happen to them as one of the most disabling aspects of the disease, seizure prediction was declared the grand challenge of epileptology [Epilepsy Foundation, 2017; Epilepsy Innovation Institute (Ei2), 2016; Kiral-Kornek et al., 2018].

At present, much effort in the development of seizure prediction algorithms has been devoted to the prediction of focal seizures, in which a local group of abnormally discharging neurons is assumed to gradually recruit a critical mass of neurons during a putative preseizure state. Results on seizure prediction performance are quite variable, with multi-variable methods taking measures of synchronization between brain structures into account usually outperforming uni-variable methods (Mormann et al., 2007). Part of this variability can be attributed to methodological shortcomings, and a list of criteria based on which prediction performance should be evaluated was established to guide good scientific practice (Mormann et al., 2007). Criteria include evaluation of prediction performance based on unselected continuous data, in-sample and out-of-sample testing with unseen (pseudo) prospective data, and evaluation with rigorous and solid statistical methods like Monte Carlo surrogate statistics to test prediction performance against chance level prediction (Mormann et al., 2007; Kuhlmann et al., 2018).

More recently developed algorithms evaluated against these criteria, employed machine learning or deep learning approaches, and were found to achieve above chance prediction (Khan et al., 2018; Kiral-Kornek et al., 2018; Eberlein et al., 2019). Both are feature extraction methods that have been proven successful in a number of pattern recognition tasks, like image and speech recognition in medical diagnosis, genomics, translation or robotics (Ratner, 2015; Daily et al., 2017; Walter et al., 2019).

Comparatively little effort has been devoted to the prediction of generalized seizures, as they have long been regarded as stochastic events (Lopes Da Silva et al., 2003). In two validated genetic rat model of absence epilepsy [rats of the WAG/Rij strain and Genetic Absence Epilepsy Rats from Strasbourg (GAERS)], characterized by generalized spike and wave discharges (SWDs) and a concomitant decrease in the level of consciousness (van Luijtelaar and Zobeiri, 2014; Depaulis and Charpier, 2018), several studies reported the presence of preictal changes in the corticothalamic system, which might be useful features for SWD prediction (Pinault et al., 2001; Polack et al., 2007; van Luijtelaar et al., 2011; Lüttjohann and van Luijtelaar, 2012; Sorokin et al., 2016). A first proof of principle for the predictability of SWDs was provided by Maksimenko et al. (2017). To achieve a measure for synchronization signaling SWD initiation, these authors calculated the product of the wavelet energy assessed in local field potential (LFP) recordings taken at three locations in the cortico-thalamic system of WAG/Rij rats. While this algorithm already reached a high sensitivity of prediction, it still suffered from a large amount of false alarms, strongly reducing the specificity of prediction.

The current study was designed to improve SWD prediction performance through (1) a systematic variation of multiple recording sites of SWDs in the cortico-thalamic system and relation to SWD prediction sensitivity and false alarm rate; (2) a thorough statistical comparison of wavelet spectra corresponding to true positive and false positive detections; and (3) training of a machine learning algorithm (random forest) to further differentiate between these two types of detections.

In line with the criteria of good scientific practice mentioned above, we assessed algorithm performance in long lasting, non-selected, pseudo-prospective 24-h recordings, taking potential diurnal variations of seizure occurrence into account (Smyk and van Luijtelaar, 2020), we incorporated in-sample and out-of-sample recordings (from two different genetic rat models of absence epilepsy, rats of the WAG-Rij strain and GAERS), and we statistically verified the results using surrogate statistics.

## Materials and Methods

### Animals, surgery, and acquisition of LFP recordings

LFP recordings of a total of 22 male WAG/Rij rats and 15 male GAERS, two well validated genetic rat models of absence epilepsy were analyzed. As both strains show several hundred spontaneously occurring SWDs per day (Depaulis and van Luijtelaar, 2006), the data are potentially suited for training and evaluation of machine learning algorithms requiring a large amount of training data.

Recordings of 16 WAG/Rij rats were taken from a previously published data set analyzing preictal network interactions in the cortico-thalamic system (Lüttjohann and van Luijtelaar, 2012, 2015). In these rats, LFP signals were simultaneously measured in freely moving animals in eight different brain structures within the cortico-thalamic system including the posterior thalamic nucleus (Po), the ventral-postero-medial thalamic nucleus (VPM), caudal and rostral part of reticular thalamic nucleus (cRTN and rRTN, respectively), anterior thalamic nucleus (ATN) as well as Layers IV, V, and VI of the somatosensory cortex (S1; coordinates are specified in Lüttjohann and van Luijtelaar, 2012). LFP signals were gathered at a constant sample rate of 2048 Hz and filtered between 1-Hz high pass (HP) and 100-Hz low pass (LP) as well as by a 50-Hz notch filter, over a period of at least 4 h. A WINDAQ-recording-system was used to digitize EEG signals (DATAQ-Instruments Inc.). Rat movement was registered via a PIR detector (RK2000DPC LuNAR PR Ceiling Mount, Rokonet RISCO Group S.A.). In additional six WAG/Rij rats LFP recordings were acquired in Layers Va, Vb, and VI of the secondary motor cortex (A/P +2.7 mm, M/L +1.2 mm, d −2.5, 2.6, 2.8 mm, respectively). Coordinates were determined relatively to bregma and according to the stereotactic atlas of Paxinos and Watson (1998).

LFP recordings of GAERS were acquired in the Münster lab. Animals aged three to nine months, born and raised at the Institute of Physiology I, Westfälische Wilhelms-University Münster, underwent stereotactic surgery under pentobarbital anesthesia (Narcoren, 50 mg/kg; Boehringer Ingelheim Vetmedica GmbH) for the implantation of recording electrodes (stainless steel, insolated with polyamide, impedance 0.1 MΩ; diameter 0.005 inch; Plastics One) in the deep Layers (IV, V, and VI) of S1 (A/P: −1.8, M/L: −3.6, d: −2.6, −2.9 −3.2). Reference and a ground electrode were placed on top of the cerebellum. Carprofen (5 mg/kg) was administered to the rats 30 min before as well as 24 and 48 h after surgery to ensure intra and postoperative analgesia.

Two weeks after surgery, animals were placed in a 43 × 28 × 42 cm Plexiglas recording box, equipped with bedding material, cage enrichment (Enviro-Dri) and free excess to food and water. Rats were connected to recording leads connected to a swivel commutator allowing LFP recordings in freely moving animals. LFP signals were amplified by an amplifier (TD 90 087, Radboud University Nijmegen, Electronic Research Group) filtered between 1 Hz (HP) and 100 Hz (LP) as well as by a 50-Hz notch filter, and digitalized with a constant sample rate of 500 Hz by WINDAQ-recording-system (DATAQ-Instruments Inc.). In addition, a PIR (Passive Infrared Registration, RK2000DPC LuNAR PR Ceiling Mount, Rokonet RISCO Group S.A.) registered rat movements. GAERS were recorded for a total of 24 h.

All experimental procedures were conducted according to the guidelines and regulations of the council of the European Union (Directive 2010/63/EU) and were approved by local authorities.

### Data processing and statistics

#### Wavelet-based SWD prediction by the Maksimenko et al. (2017) algorithm, comparison between combinations of recording sites in the cortico-thalamic system

In an attempt to determine the optimal recording sites for SWD prediction and to gain additional insight into network interactions in the cortico-thalamic system in relation to the generation of SWD, we assessed SWD prediction performance in all possible combinations of two and three different recording sites in the cortico-thalamic system (Table 1), using the algorithm previously published by Maksimenko et al. (2017).

**Table 1:** Combinations of recording sites analyzed by the Maksimenko et al. algorithm and achieved average sensitivities of prediction and false alarm rates

Number of simultaneous recording sites	Combination number	Area 1	Area 1	Area 3	Abbreviation in text and figures	Average sensitivity	Average nFP/h
3	1	ctx 4	ctx 5	ctx 6	CCC	61,755	85,962
	2	ctx 4	ctx 5	Po	CCT	48,392	65,199
	3	ctx 4	ctx 5	ATN	CCT	45,974	85,363
	4	ctx 4	ctx 5	rRTN	CCT	44,230	69,947
	5	ctx 4	ctx 5	cRTN	CCT	50,600	58,431
	6	ctx 4	ctx 5	VPM	CCT	46,007	61,826
	7	ctx 4	ctx 6	Po	CCT	50,718	68,470
	8	ctx 4	ctx 6	ATN	CCT	48,932	82,776
	9	ctx 4	ctx 6	rRTN	CCT	45,690	71,436
	10	ctx 4	ctx 6	cRTN	CCT	51,823	60,125
	11	ctx 4	ctx 6	VPM	CCT	50,269	58,889
	12	ctx 5	ctx 6	Po	CCT	48,880	79,424
	13	ctx 5	ctx 6	ATN	CCT	48,345	95,587
	14	ctx 5	ctx 6	rRTN	CCT	51,354	65,995
	15	ctx 5	ctx 6	cRTN	CCT	48,963	72,081
	16	ctx 5	ctx 6	VPM	CCT	48,708	62,217
	17	ctx 4	Po	ATN	CTT	36,121	98,180
	18	ctx 4	Po	rRTN	CTT	35,171	97,276
	19	ctx 4	Po	cRTN	CTT	35,430	95,410
	20	ctx 4	Po	VPM	CTT	34,470	99,526
	21	ctx 4	ATN	rRTN	CTT	38,536	82,294
	22	ctx 4	ATN	cRTN	CTT	34,133	101,225
	23	ctx 4	ATN	VPM	CTT	32,981	99,376
	24	ctx 4	rRTN	cRTN	CTT	35,892	93,150
	25	ctx 4	rRTN	VPM	CTT	33,018	101,039
	26	ctx 4	cRTN	VPM	CTT	37,588	83,424
	27	ctx 5	Po	ATN	CTT	38,046	96,665
	28	ctx 5	Po	rRTN	CTT	36,549	93,522
	29	ctx 5	Po	cRTN	CTT	36,114	97,002
	30	ctx 5	Po	VPM	CTT	34,702	99,814
	31	ctx 5	ATN	rRTN	CTT	40,655	77,191
	32	ctx 5	ATN	cRTN	CTT	36,485	98,925
	33	ctx 5	ATN	VPM	CTT	33,716	98,891
	34	ctx 5	rRTN	cRTN	CTT	37,172	90,429
	35	ctx 5	rRTN	VPM	CTT	33,526	100,798
	36	ctx 5	cRTN	VPM	CTT	38,023	82,687
	37	ctx 6	Po	ATN	CTT	40,751	95,255
	38	ctx 6	Po	rRTN	CTT	38,563	93,038
	39	ctx 6	Po	cRTN	CTT	38,292	95,827
	40	ctx 6	Po	VPM	CTT	36,516	101,606
	41	ctx 6	ATN	rRTN	CTT	43,434	72,403
	42	ctx 6	ATN	cRTN	CTT	37,946	100,546
	43	ctx 6	ATN	VPM	CTT	35,950	98,257
	44	ctx 6	rRTN	cRTN	CTT	40,527	84,918
	45	ctx 6	rRTN	VPM	CTT	35,363	100,356
	46	ctx 6	cRTN	VPM	CTT	38,784	87,363
	47	Po	ATN	rRTN	TTT	35,880	103,088
	48	Po	ATN	cRTN	TTT	31,342	115,496
	49	Po	ATN	VPM	TTT	30,849	115,094
	50	Po	rRTN	cRTN	TTT	33,263	109,348
	51	Po	rRTN	VPM	TTT	31,252	116,632
	52	Po	cRTN	VPM	TTT	30,485	116,111
	53	ATN	rRTN	cRTN	TTT	36,646	89,893
	54	ATN	rRTN	VPM	TTT	34,497	98,061
	55	ATN	cRTN	VPM	TTT	30,137	110,576
	56	rRTN	cRTN	VPM	TTT	30,907	115,390
	57	Mctx 5a	Mctx 5b	Mctx 6	MCCC	33,330	129,803
2	1	ctx 4	ctx 5		CC	31,173	211,365
	2	ctx 4	ctx 6		CC	34,619	209,386
	3	ctx 5	ctx 6		CC	33,612	242,989
	4	ctx 4	VPM		CT	21,408	123,705
	5	ctx 4	ATN		CT	20,799	148,887
	6	ctx 4	Po		CT	21,987	151,854
	7	ctx 4	cRTN		CT	23,729	122,750
	8	ctx 4	rRTN		CT	25,276	130,967
	9	ctx 5	VPM		CT	23,357	120,332
	10	ctx 5	ATN		CT	22,728	158,520
	11	ctx 5	Po		CT	24,474	151,471
	12	ctx 5	cRTN		CT	24,874	130,418
	13	ctx 5	rRTN		CT	29,267	121,645
	14	ctx 6	VPM		CT	23,514	146,704
	15	ctx 6	ATN		CT	24,906	174,084
	16	ctx 6	Po		CT	25,886	171,314
	17	ctx 6	cRTN		CT	25,948	145,599
	18	ctx 6	rRTN		CT	31,349	137,519
	19	VPM	ATN		TT	10,411	157,414
	20	VPM	Po		TT	10,741	186,945
	21	VPM	cRTN		TT	14,999	151,043
	22	VPM	rRTN		TT	15,703	155,311
	23	ATN	Po		TT	12,648	179,928
	24	ATN	cRTN		TT	10,670	165,252
	25	ATN	rRTN		TT	20,339	142,317
	26	Po	cRTN		TT	11,267	171,176
	27	Po	rRTN		TT	17,575	166,227
	28	cRTN	rRTN		TT	21,339	142,157

ctx4, Layer IV of S1; ctx5, Layer V of S1; ctx6, Layer VI of S1; ATN, anterior thalamic nucleus; VPM, vertral-postero-medial thalamic nucleus; Po, posterior thalamic nucleus; rRTN, rostral reticular thalamic nucleus; cRTN, caudal reticular thalamic nucleus; Mctx5a, Layer Va of secondary motor cortex; Mctx5b, Layer Vb of secondary motor cortex; Mctx6, Layer VI of secondary motor cortex.

For SWD prediction, the Maksimenko et al. (2017) algorithm determines in each LFP trace, the mean wavelet energy within a time window of 500 ms shifting along the complete LFP trace sample by sample. In each trace (1) and at each time step (t), the wavelet energy (W) within the frequency range of 5–10 Hz corresponding to the precursor [W_(5–10 Hz)_(t)] is calculated using wavelet transformation with a modified Morlet mother function (van Luijtelaar et al., 2016; Maksimenko et al., 2017). This energy obtained in each trace is multiplied to determine the occurrence of cortico-thalamic synchronization at each moment in time [W_(5–10 Hz)_(t) = W1_(5–10 Hz)_(t) × W2_(5–10 Hz)_(t) × W3_(5–10 Hz)_(t)]. Moreover, wavelet energy is calculated and multiplied in each channel for a frequency range of 3–5 Hz in accordance to the light slow wave sleep [W_(3–5 Hz)_(t) = W1_(3–5 Hz)_(t) × W2_(3–5 Hz)_(t) × W3_(3–5 Hz)_(t)] and within a frequency range of 7–20 Hz representing sleep spindles [W_(7–20 Hz)_(t) = W1_(7–20 Hz)_(t) × W2_(7–20 Hz)_(t) × W3_(7–20 Hz)_(t); Fig. 4*A*].

Decision on whether a SWD precursor is present is based on three criteria: (1) energy of W_(5–10 Hz)_(t) must exceed an individualized specific threshold; (2) energy of W_(5–10 Hz)_(t) must exceed energy of W_(3–5 Hz)_(t); (3) energy of W_(5–10 Hz)_(t) must exceed energy of W_(7–20 Hz)_(t).

For determination of optimal recording sites for SWD prediction, LFP recordings (duration 4 h), simultaneously obtained within the cortico-thalamic system in GAERS and WAG/Rij rats, were fed into the wavelet-based SWD prediction algorithm of Maksimenko et al. (2017), testing data from the various recordings sites in all possible combinations (Table 1). For WAG/Rij rats, a total number of 57 combinations composed of LFP recordings from three recording sites and 28 combinations, composed of LFP recordings from two recording sites (see Table 1), were presented to the algorithm. For GAERS, data from three recording sites in Layers IV, V, and VI of S1 were used. Each combination of recording sites can be found in Table 1; C, T, M globally refers to recording sites in S1, thalamus, and secondary motor cortex, respectively.

Since SWD prediction quality depends on the above-mentioned individualized threshold, SWD prediction performance of each recording site combination was determined for a total of 14 fixed threshold values ranging from 0.1 to 0.75 for all combinations of three recording sites, and a total of 16 fixed threshold values ranging from 0.005 to 0.04 for all combinations composed of two recording sites. Of note, the difference in magnitude in the threshold values for two and three recording sites is attributed to the fact that detection relies on the product of either two or three wavelet energy values (see above). For prediction based on two *versus* three recording sites, the outer threshold levels (minimum and maximum) correspond to saturated levels of either sensitivity or false alarm rates for all tested combinations.

**Figure 1.**
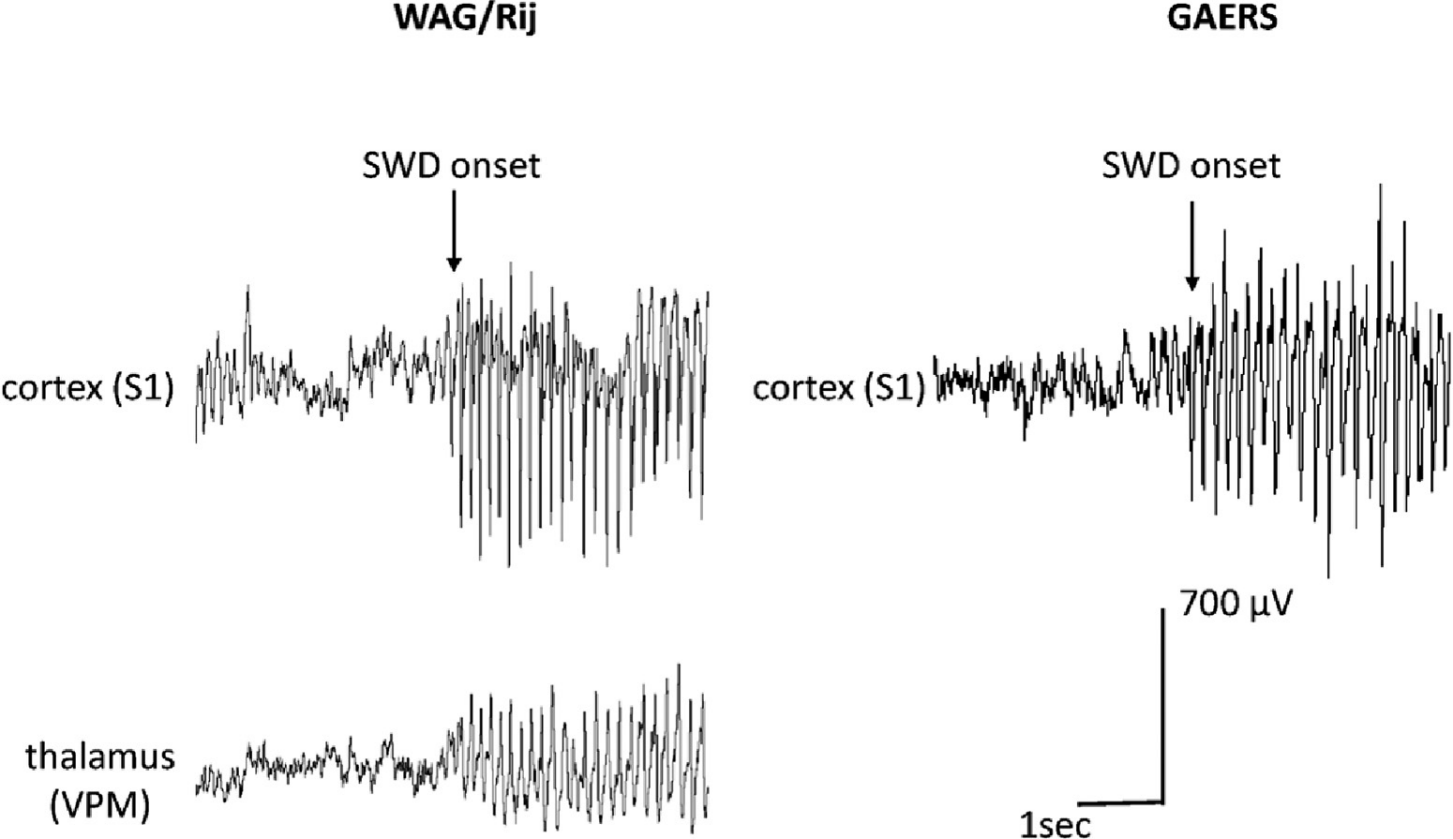
Exemplary LFP recordings in the deep S1 of a GAERS (right) as well as simultaneously recorded LFPs in the deep S1 and VPM of a WAG/Rij rat (upper left panel and lower left panel, respectively). Arrows indicates the onset of the SWD, determined according to the criteria outlined by van Luijtelaar and Coenen (1986), taking the peak of the first spike of twice the background as reference for SWD onset.

Detections of the algorithm occurring within a 1s preictal period before SWD onset were regarded as true positives, while detections at interictal time points were regarded as false positives. SWD onset was determined according to the criteria outlined by van Luijtelaar and Coenen (1986), taking the peak of the first spike of twice the amplitude of the background EEG as a reference to mark the onset of the SWD (Fig. 1). In case of differences in spike timing between recording sites, notably occurring in the range of milliseconds (Lüttjohann and van Luijtelaar, 2012), the peak of the first spike earliest in time was taken as SWD onset.

For each combination of recording sites, and for each of the threshold values, the sensitivity [sensitivity = number of correctly predicted SWDs/(number of correctly predicted SWDs + number of unpredicted SWDs) × 100%] of SWD prediction as well as the false alarm rate were determined.

Linear regression analysis (Pearson correlation) was used to determine the degree of interdependence between the sensitivity of prediction and false alarm rate.

Statistical comparison of sensitivity and false alarm rate between different combinations of recording sites were performed using ANOVA with sensitivity or false alarm rate as dependent variable, combination of recording sites as between subject factor 1, number or recording sites (2, 3) as between subject factor 2, threshold as covariate 1 and false alarm rate or sensitivity as covariate 2.

To avoid multiple comparison problems all combinations of recording sites were grouped for *post hoc* analyses as follows: (1) two intracortical recording sites in S1 (CC); (2) one cortical recording site in S1 and one thalamic recording site (CT); (3) two intrathalamic recording sites (TT); (4) three intracortical recording sites in S1 (CCC); (5) two cortical recording sites in S1 and one thalamic recording site (CCT); (6) one cortical recording site in S1 and two thalamic recording sites (CTT); (7) three intrathalamic recording sites (TTT); and (8) three intracortical recording sites in the secondary motor cortex (MCCC). *Post hoc* analyses included: ANOVA with sensitivity or false alarm rate as dependent variable, group of channel combinations (CC, TC, TT, CCC, CCT, CTT, TTT, MCCC) as between subject factor 1, number of recording sites (2, 3) as between subject factor 2, threshold as covariate 1 and false alarm rate or sensitivity as covariate 2.

All statistical analyses were performed using IBM SPSS version 25. Data are expressed as the arithmetic mean values ± SEM. Differences were considered statistically significant when *p* ≤ 0.05 (*), *p* ≤ 0.01 (**), and *p* ≤ 0.001 (***).

#### Comparison of wavelet spectra corresponding to true positive and false positive predictions

Irrespective of the combination of recording sites, the Maksimenko et al. (2017) algorithm results in relatively high false alarm rates. Therefore, we determined preexisting differences in spectra corresponding to either true positive or false positive predictions. Wavelet spectra of all true positive detections, and a total number of 50 randomly selected false positive detections, as identified by the algorithm of Maksimenko et al. (2017), were calculated from LFP traces acquired in the deep Layers (IV, V, and VI) of S1 in GAERS and WAG/Rij rats. Time point zero indicates the time point of precursor detection at the end of a 500-ms analysis window (ranging from −0.5 to 0), in which either the true positive precursor or the false positive was detected.

Average wavelet energy within different frequency bands was statistically compared between true and false detections using repeated measures ANOVA with average wavelet energy as dependent variable, type of detection (true positive, false positive) as within subjects factor 1, frequency band (W_(5–10 Hz)_, W_(3–5 Hz)_, and W_(7–20 Hz)_) as within subjects factor 2 and rat strain (GAERS, WAG/Rij rats) as between subject factor.

#### Random forest machine learning algorithm for differentiation between true positive and false positive predictions

In an attempt to further differentiate between true and false positive predictions, we trained a random forest machine learning algorithm. The wavelet energy extracted for true and false detections was fed into a random forest (Birjandtalab et al., 2017) consisting of a total of 1000 decision trees. Different numbers of trees were experimentally varied to investigate the effect of forest size on classification performance. For each true and false positive prediction produced by the Maksimenko et al. (2017) algorithm, nine wavelet energy values corresponding to the values assessed in the three frequency bands (W_(5–10 Hz)_, W_(3–5 Hz)_, and W_(7–20 Hz)_) at three different recording sites, were presented to the algorithm to extract features for classification. Majority voting of the different trees in the random forest yielded final classification.

Training of the random forest was performed with spectra obtained in 70% of all recorded data in six WAG/Rij rats and six GAERS, and classification performance was evaluated on the remaining 30% of unseen data of the same rats (i.e., in-sample testing). As epileptic seizures or preictal events are underrepresented compared with the vast number of interictal fragments or false positive predictions, a random undersampling approach was taken in a first step to create a balanced training set and thereby ensure balanced learning (Kubat et al., 1997). All true positive detections were fed into the algorithm, matched by an equal number of randomly selected false positive detections. In this way, a total of 100 random forest were trained. Of note, each random forest was fed with a different set of false positive detections. Obtained results correspond to the performance of a single trained random forest, which was found to reach an average performance of these 100 trained trees.

In order to allow an unbiased comparison of classification performance of the random forest between different combinations of recording sites, we adjusted the detection threshold of the Maksimenko et al. (2017) algorithm for each combination to reach a 60% sensitivity of SWD prediction for the extraction of the time points and wavelet features for training and evaluation of classification.

To assess the classification performance of the random forest the balanced accuracy of classification was calculated as (sensitivity of classification + specificity of classification)/2, with specificity = (number of false positives predicted as false positives/(number of false positives predicted as false positives + number of false positives predicted as true positives)) × 100% and sensitivity = (number of true positives predicted as true positives/(number of true positives predicted as true positives + number of true positives predicted as false positives)) × 100%. Moreover an F1 score defined as F1 = 2 × ((precision × sensitivity)/(precision + sensitivity)) × 100% was calculated, where precision equals (number of true positives predicted as true positives/(number of true positives predicted as true positives + number of false positives predicted as true positives)) × 100%.

Classification performance of the random forest was compared with ANOVA between the different groups of recording sites in WAG/Rij rats: (1) recordings in Layers V and VI of S1, referred to as CC (*n* = 145); (2) recordings in Layers IV, V, and VI of S1, referred to as CCC (*n* = 161); (3) recordings in Layers IV and VI of S1 and VPM, referred to as CCT (*n* = 161); (4) recordings in Layer VI of S1, VPM, and RTN, referred to as CTT (*n* = 161); (5) recordings in VPM, cRTN, and Po, referred to as TTT (*n* = 145); and (6) recordings in Layers Va, Vb, and VI of secondary motor cortex, referred to as MCCC (*n* = 161). In addition, classification performance was assessed in recordings from Layers IV, V, and VI of S1 in GAERS, referred to as GCCC (*n* = 145, *n* = 161, *n* = 1844) and compared with results achieved in WAG/Rij rats using ANOVA. Furthermore, classification performance of each group was evaluated against chance level using surrogate statistics (see below).

#### Probing the random forest machine learning algorithm for maximal SWD prediction performance

Next, the random forest machine learning combined with the Maksimenko et al. (2017) algorithm was probed for maximal prediction performance of SWD. Wavelet features for true and false predictions were extracted in LFP recordings obtained in the deep Layers (IV, V, and VI) of S1 of six GAERS at a threshold value reaching a 90% sensitivity for SWD prediction, and were used for training and in-sample testing as described above. Moreover, performance of random-forests trained in this approach were assessed in unseen 24-h recordings from a separate group of nine GAERS rats (out-of-sample testing).

For in-sample testing and out-of-sample testing, performance was statistically evaluated against chance level prediction using surrogate statistics. To this end, training data of true and false detections were randomly assigned to the two classes (total of 1000 randomizations), and for each randomization the average balanced accuracy achieved in the unseen data were determined and displayed in a histogram. In case the achieved balanced accuracy computed for the random forest trained with the real (i.e., non-randomized) training data were positioned above the 95th quantile of the histogram, algorithm performance was regarded as significant above chance level.

Lastly, as classification performance of the random forest was found to be reduced in the out-of-sample testing, likely resulting from an insufficient amount of false positive predictions presented to the algorithm during training, a separate set of random forests (*n* = 100) was trained in a (moderate) oversampling approach. A multiple (4) of all true positive predictions and a matched number of randomly selected false positive predictions, derived in LFP recordings of the deep Layers (IV, V, and VI) of S1 in six GAERS at a threshold value of 90%, were used to train the random forests. Determination of an appropriate oversampling factor was performed by comparison of classification performances achieved at different oversampling factors, ranging between 2 and 7. Higher rates of oversampling were omitted to avoid overtraining. As for the under-sampling approach, classification performance was assessed in unseen 24-h recordings from a separate group of 9 GAERS rats (out-of-sample testing) and tested against chance level using surrogate statistics (see above).

Performance presented in the results corresponds to the performance of a single trained random forest, reaching an average performance of these 100 trained trees.

### Histology

At the end of the recordings, a direct current (9 V, 25 μA, 2 s in duration) was pathed though each electrode to create an electrolytic lesion at the location of the tip of the electrode. Animals were killed with an intraperitoneal injection of pentobarbital (Narcoren, 150 mg/kg; Merial GmbH). The brain was quickly removed and placed in a 4% paraformaldehyde (PFA) solution for at least 24 h. Brains were fixated in a 30% sucrose solution and cut into 60 μm slices with the aid of a microtome. Slices were mounted on microscope slides, stained with cresyl violet, and inspected under a light-microscope (dnt, DigiMicro Profi) for identification of the microlesions. Recording sites were extrapolated from the center of the lesion relative to cortical depth and neighboring cortical layers. Only recordings from verified recording positions were included in the analysis.

### Code accessibility

The random forest algorithm was programmed in Python and requires previous installation of Python for execution. The code of the random forest algorithm is available as Extended Data 1.

10.1523/ENEURO.0160-21.2021.ed1 Extended Data 1 RF oversampling seizure prediction. Download Extended Data 1, ZIP file.

## Results

### Electrophysiological characteristics of SWDs in GAERS and WAG/Rij rats

Exemplary LFP recordings of GAERS and WAG/Rij rats are displayed in Figure 1. LFP signals of GAERS, recorded for 24 h, displayed frequent (average of 17 per hour) SWDs of 10- to 30-s duration at a main frequency of 5–7 Hz. Occurrence of SWDs showed the well documented diurnal variation with highest rates of occurrence at the beginning of the dark phase and lowest rates of occurrence at beginning of the light phase (Smyk and van Luijtelaar, 2020). LFP signals in WAG/Rij rats were acquired during 4 h of the dark phase. WAG/Rij rats showed on average 10 SWDs per hour, with a mean duration of 7 s and a slightly higher internal frequency of 8–10 Hz. Spikes in thalamus typically possessed a smaller amplitude (500 vs 700 μV) and broader form, with a reversed polarity as compared with those in cortex. All differences of SWD morphology between strains (i.e., different internal frequency) and recording sites (i.e., amplitude, polarity and sharpness of spike) are in accordance with previously published data (Sitnikova and van Luijtelaar, 2007; Akman et al., 2010; Lüttjohann and van Luijtelaar, 2012).

### Influence of cortico-thalamic recording sites on SWD prediction performance

In a first set of experiments, we sought to identify the influence of LFP recording sites on SWD prediction performance. LFP recordings were simultaneously obtained at multiple sites in the cortico-thalamic system of WAG/Rij rats, specifically in the deep Layers (IV, V, and VI) of the S1, secondary motor cortex, and thalamic nuclei VPM, Po, ATN, rRTN, and cRTN.

Recordings from either two or three sites in all possible combinations (yielding a total of 85 combinations) were fed into the wavelet-based algorithm (Maksimenko et al., 2017). Sensitivity and false alarm rate of the algorithm were compared in these 85 combinations (Table 1). For *post hoc* analysis combinations were grouped as either CC (two intracortical recording sites in S1), CT (one cortical recording site in S1 and one thalamic recording site), TT (two intrathalamic recording sites, CCC (three intracortical recording sites in S1), CCT (two cortical recording sites in S1 and one thalamic recording site), CTT (one cortical recording site in S1 and two thalamic recording sites), TTT (three intrathalamic recording sites), or MCCC (three intracortical recording sites in the secondary motor cortex), respectively. Moreover, SWD prediction performance of each combination of recording sites was determined at multiple threshold values employed for precursor detection. As ANOVA revealed a significant influence of threshold on both sensitivity of prediction (*F*_(1,10980)_ = 3995, *p* < 0.001, *R*^2^ = 0.26; the higher the threshold, the lower the sensitivity) and false alarm rate (*F*_(1,10980)_ = 10.7, *p* < 0.05, *R*^2^ = 0.1; the higher the threshold, the lower the false alarm rate), threshold was taken as a covariate factor into statistical analysis to allow comparison of prediction performance between different combinations of recording sites regardless of any possible threshold effects.

**Figure 2.**
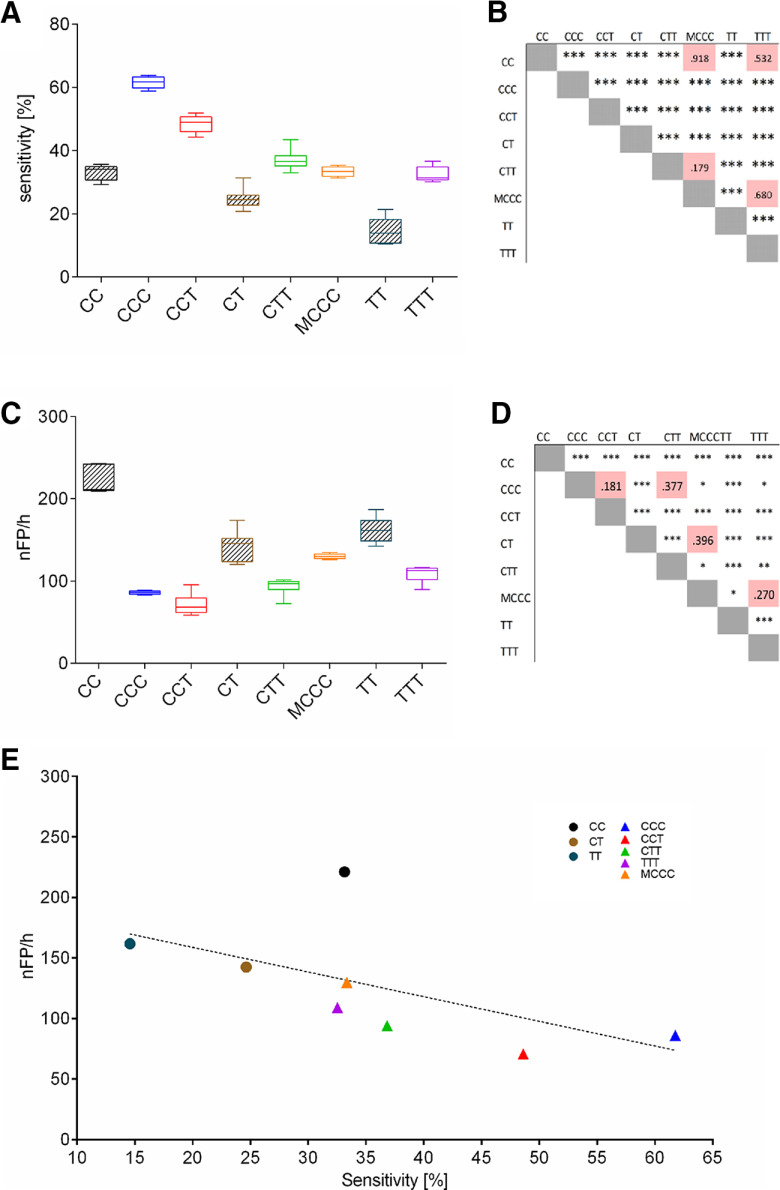
Wavelet analysis for SWD prediction. Relative sensitivity (***A***) and average false alarm rate (***C***) of SWD prediction for different combinations of recording sites in the cortico-thalamic system, obtained by the Maksimenko et al. (2017) algorithm. LFPs, simultaneously recorded in the cortico-thalamic system of WAG-Rij rats, were analyzed in combinations of either two or three recording sites. Results from all 85 combinations are presented in Table 1. To avoid Type II errors, all combinations of recording sites were grouped as either CC (two intracortical recording sites in S1), CT (one cortical recording site in S1 and one thalamic recording site), TT (two intrathalamic recording sites), CCC (three intracortical recording sites in S1), CCT (two cortical recording sites in S1 and one thalamic recording site), CTT (one cortical recording site in S1 and two thalamic recording sites), TTT (three intrathalamic recording sites), or MCCC (three intracortical recording sites in the secondary motor cortex), respectively. ***B***, ***D***, Results of *post hoc* comparison verified by ANOVA, with *** indicating significance at a *p* < 0.001 level and * indicating significance at a *p* < 0.05 level, for sensitivity of prediction (***B***) and false alarm rate (***D***), respectively. ***E***, Relationship of false alarm rates and average sensitivity of SWD prediction for different combinations of recording sites in the cortico-thalamic system of WAG/Rij rats, analyzed by the Maksimenko et al. (2017) algorithm. Note highest sensitivity with a low false alarm rate for prediction based on three intracortical recordings in S1 (blue triangle) that outperforms all other combinations of recording sites. Further note the negative correlation between both indicators of SWD prediction performance (*r* = −0.716; *p* < 0.001), indicating that higher SWD prediction sensitivity at any given combination of recording sites does not occur at the trade-off of a high false alarm rate.

ANOVA revealed significant differences in both the achieved sensitivity of prediction as well as the produced false alarm rate between the different combinations of recording sites (*F*_senitivity(84,10980)_ = 13.47, *p* < 0.001, *R*^2^ = 0.37; *F*_nFP(84,10980)_ = 2.47, *p* < 0.001, *R*^2^ = 0.1; Fig. 2; Table 1).

On average, predictions based on three recording sites reached significantly higher sensitivities (Fig. 2*A*; Table 1) and lower false alarm rates (Fig. 2*C*; Table 1) as compared with predictions based on two recording sites (*F*_senitivity(1,10980)_ = 935.7, *p* < 0.001, *R*^2^ = 0.07; *F*_nFP(1,10980)_ = 116.3, *p* < 0.001, *R*^2^ = 0.02).

Regarding the false alarm rate (Fig. 2*C*; Table 1) predictions based on three intracortical recordings in S1 (CCC) and predictions based on cortico-thalamic recording sites (CCT and CTT) showed a significantly smaller number of false alarms compared with predictions based on three intrathalamic recordings (TTT; all *p* < 0.001; average false alarm rate of CCC = 85.2 ± 10.6, CTT= 94.7 ± 3.0, CCT= 70.6 ± 3.5 and TTT = 110.2 ± 5.4). Predictions based on three intracortical recordings acquired in the secondary motor cortex (MCCC), on the other hand, resulted in significantly more false alarms (average false alarm rate MCCC = 129.8 ± 17.9) as compared with predictions based on CCC, CCT and CTT combinations (all *p* < 0.05). Highest false alarm rates with an average of 221.1 ± 6.2 were found for predictions based on two intracortical recordings acquired in S1 (all *p* < 0.001).

Regarding the sensitivity of SWD prediction, predictions based on recordings in Layers IV, V, and VI of S1 significantly outreached all other combinations with an average sensitivity of 61.7 ± 1.5% (all *p* < 0.001; Fig. 2*A*; Table 1).

Among the remaining combinations with three recording sites, MCCC, TTT and CTT showed significantly lower sensitivities compared with predictions based on two recording sites in S1 combined with one thalamic site (CCT; all *p* < 0.001; Fig. 2*A*,*B*; Table 1). Lowest sensitivity was reached for predictions on two thalamic recordings (average sensitivity TT = 13.7 ± 0.8%), while predictions based on two cortical recording sites in S1 reached a medium sensitivity of 33.0 ± 0.9% (Fig. 2*A*,*B*; Table 1).

To estimate the degree of interdependence between achieved sensitivity of SWD prediction and resulting false alarm rate regression analysis was performed. Analysis revealed a significant negative correlation between both indicators of SWD prediction performance (*r* = −0.716; *p* < 0.001; Fig. 2*E*), indicating that a higher SWD prediction sensitivity, achieved for a given combination of recording sites, does not occur at the trade-off of a high false alarm rate. The same clusters as described above could be identified in the regression pattern including higher false alarm rates and lower sensitivities for predictions on two recording sides within the cortico-thalamic system, highest false alarm rate and medium sensitivity for predictions based of two intracortical recordings in S1, medium sensitivity and medium false alarm rate for predictions based on three intracortical recordings in M2 and highest sensitivity with a low false alarm rate for prediction based on three intracortical recordings in S1 (Fig. 2). Of note, regardless of recording site combination, algorithm performance remained at a low level including only moderate sensitivities of SWD prediction and high false alarm rates.

### Out-of-sample testing: comparison between rat strains

**Figure 3.**
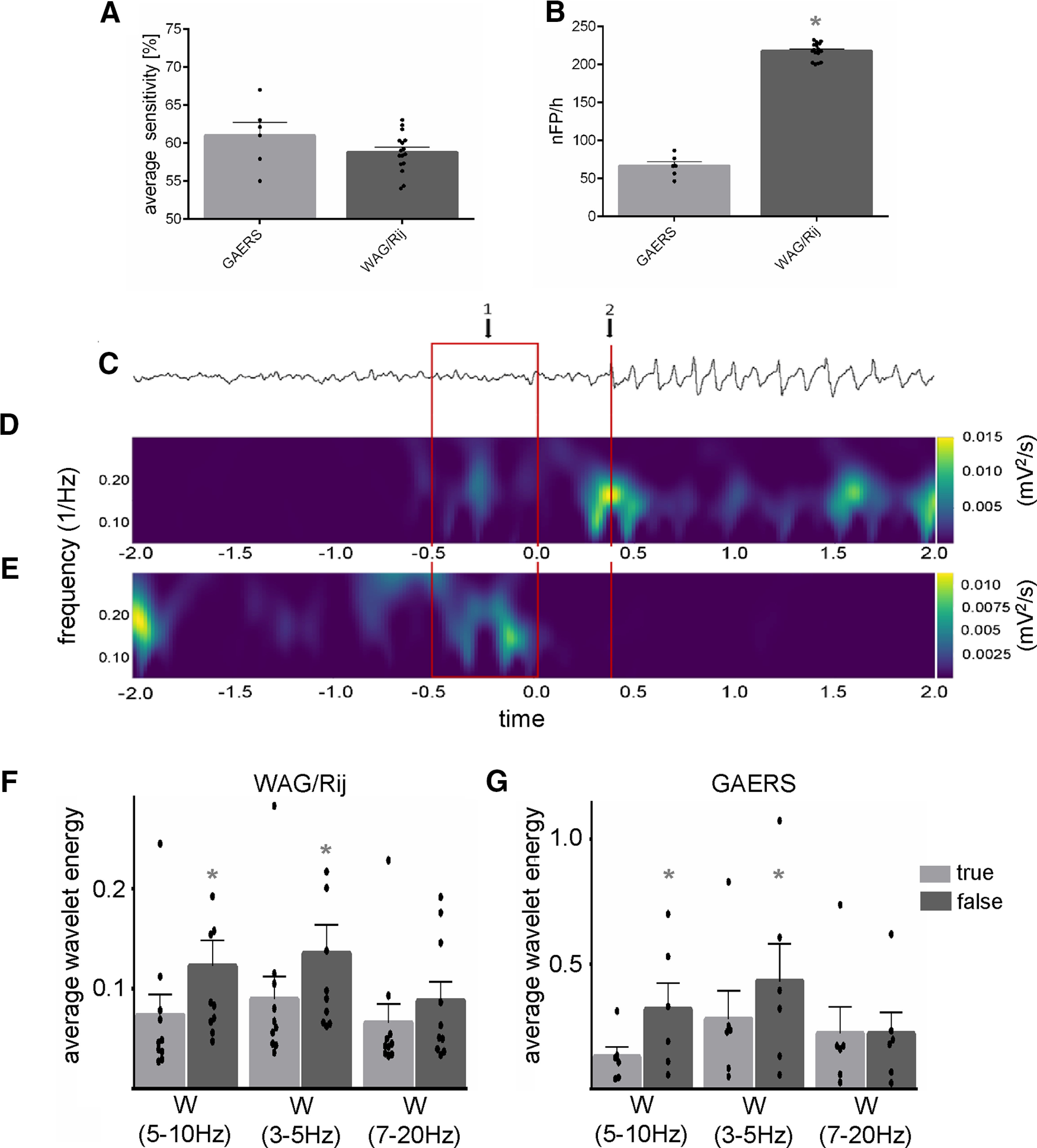
SWD prediction in two genetic rat models of absence epilepsy. ***A***, ***B***, Average sensitivity of SWD prediction (***A***) and false alarm rate expressed in number of false positives per hour (nFP/h; ***B***) achieved by the Maksimenko et al. (2017) algorithm assessed in 4-h lasting LFP recordings, obtained in Layers IV, V, and VI of S1 in GAERS and WAG/Rij rats. ***C–E***, Comparison of wavelet spectra of true and false positive predictions. An exemplary LFP trace depicting a pre-SWD -> SWD transition is presented in ***C***. Onset of SWD is marked by red vertical line termed 2. The corresponding spectrogram of a true positive detection identified in intracortical LFP recordings in S1 of a GAERS is shown in ***D***. Time period −0.5–0 (red rectangle termed 1) features the analysis window (window size 500 ms) in which the true positive precursor is detected. An exemplary spectrogram of a false positive detection is shown in ***E***. Again, Time period −0.5–0 features the analysis window (window size 500 ms) in which the false positive precursor is detected. ***F***, Statistical comparison of the product of wavelet energy, assessed in the frequency bands W_(5–10 Hz)_, W_(3–5 Hz)_, and W_(7–20 Hz)_ (Maksimenko et al., 2017), between true and false positives in WAG/Rij rats. ***E***, Statistical comparison of the product of wavelet energy, assessed in the frequency bands W_(5–10 Hz)_, W_(3–5 Hz)_, and W_(7–20 Hz)_ (Maksimenko et al., 2017), between true and false positives in GAERS; * indicates a significant difference verified by ANOVA at level of * *p* < 0.05.

Both, GAERS and WAG/Rij rats are well validated genetic rat models of absence epilepsy sharing genetic, physiological, and behavioral characteristics (Depaulis and van Luijtelaar, 2006), although slight, but significant, differences in electrophysiological parameters of SWDs have been reported (Akman et al., 2010). Therefore, we evaluated the prediction performance of the Maksimenko et al. (2017) algorithm also in GAERS. Prediction performance was assessed in 4-h lasting LFP recordings, obtained in Layers IV, V, and VI of S1 in GAERS and WAG/Rij rats, and was compared between the two strains. Significant differences between rat strains were revealed for the produced false alarm rate, with significantly more false alarms in WAG/Rij rats compared with GAERS (*p* < 0.001; Fig. 3*B*). On the other hand, no significant differences were seen between GAERS and WAG/Rij rats for the sensitivity of prediction (*p* > 0.05; Fig. 3*A*).

### Comparison of true and false positive detections

Irrespective of the combination of recording sites, the Maksimenko et al. (2017) algorithm resulted in relatively high false alarm rates. Therefore, we determined preexisting differences in spectra corresponding to either true positive or false positive predictions in a next experimental step.

Figure 3*D*,*E* depicts exemplary spectrograms of true and false positive SWD predictions, respectively. Time period −0.5–0 features the analysis window (window size 500 ms) in which either the true positive precursor or the false positive was detected. The onset of the SWD is depicted at time point 0.4 s on the *x*-axis (Fig. 3*C*,*D*). At this point a strong increase in the product of the wavelet energy can be noted in the main frequency band of the SWD (i.e., 5–10 Hz). On average, precursor activity ∼900–300 ms before SWD onset.

Next, the product of wavelet energy, assessed in the frequency bands W_(5–10 Hz)_, W_(3–5 Hz)_, and W_(7–20 Hz)_ (Maksimenko et al., 2017), was statistically compared between true and false positives across the two rat strains. Data revealed significant differences between true and false positives in the frequency bands W_(5–10 Hz)_ and W_(3–5 Hz)_. False positives possessed a higher wavelet-energy product as compared with true-positives (all *p* < 0.05). For both frequency bands, this difference was significantly more pronounced in GAERS compared with WAG/Rij rats (*F*_(2,28)_ = 7.3, *p* < 0.05, *R*^2^ = 0.3; Fig. 3*F*,*G*).

### A random forest machine learning algorithm for improvement of SWD prediction

**Figure 4.**
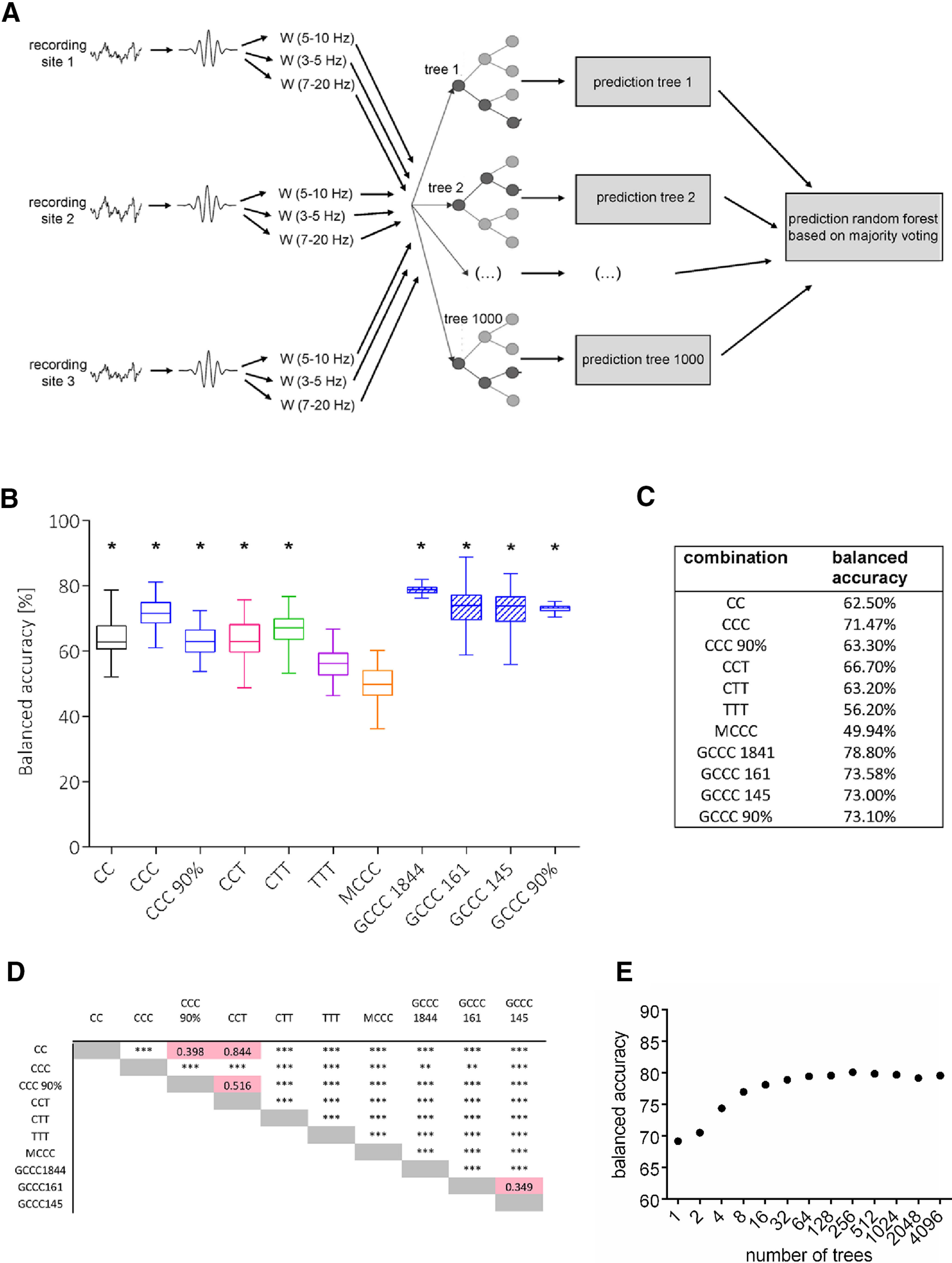
Differentiation between true and false positives by a random forest machine learning algorithm. ***A***, Schematic representation of the random forest machine learning algorithm for differentiation between true positive and false positive predictions. After wavelet analysis of either two or three simultaneously recorded LFP traces, the wavelet energies (W_(5–10 Hz)_, W_(3–5 Hz)_, and W_(7–20 Hz)_) extracted in each trace are fed to a random forest composed of 1000 decision trees. Final classification of the random forest is yielded from a majority voting of the different trees. ***B***, Out-of-sample performance (expressed as balanced accuracy) of random forests. Training in an undersampling approach on wavelet spectra derived from recordings in Layers V and VI of S1 (CC); recordings in Layers IV, V, and VI of S1 (CCC); recordings in Layers IV and VI of S1 and VPM (CCT); recordings in Layer VI of S1, VPM, and RTN (CTT); recordings in VPM, cRTN, and Po (TTT) of WAG/Rij rats at a sensitivity of 60%; and recordings in Layers IV, V, and VI of S1 of GAERS at a sensitivity of 60% (GCCC) or 90% (GCCC90%). Numbers in GAERS groups (1844, 161, 145) refer to the different amount of true/false positive fragments, with which the random forest was trained. Stars in ***B*** indicate a significant classification above chance as validated by surrogate statistics with * indicating significance at a *p* < 0.05 level. ***C***, Table of achieved average balanced accuracies achieved by analysis of the different combinations of recording sites. ***D***, Statistics between group comparison of balanced accuracies performed with ANOVA with * indicating significance at a *p* < 0.05, **p < 0.01, and ***p < 0.001 level. ***E***, Relation between classification accuracy and the number of incorporated trees in the random forest.

Since significant differences in the wavelet spectra of true and false positives were revealed, a random forest machine learning algorithm was trained to differentiate between true positive and false positive detections. In a first step, a random undersampling approach was used to create a training data set. Here, true positives detected in 70% of recordings from six WAG/Rij or six GAERS rats and an equal amount of randomly selected false positives derived from 70% of recordings in the same rats were used as training data. For in-sample performance evaluation, the algorithm was confronted with the remaining 30% of unseen data (for more details, see Materials and Methods). As in the paragraphs above, classification performance of the random forest was compared between different combinations of recording sites in WAG/Rij rats and between rat strains (Fig. 4).

In WAG/Rij rats, classification performance of the random forest was significantly above chance level for all combinations of recording sites (average balanced accuracy CCC = 71.5%, CCT = 66.7%, CTT = 63.2%, CC = 62.5%; all *p* < 0.05) except for spectra derived from three intrathalamic recording sites (average balanced accuracy TTT = 56.2%; *p* > 0.05) and spectra derived from recordings in Layers Va, Vb, and VI of the secondary motor cortex (average balanced accuracy MCCC = 49.9%; *p* > 0.05; Fig. 4*B*). Highest classification accuracies were derived from three intracortical recordings acquired in S1, as was seen using the Maksimenko algorithm above (all *p* < 0.05). Classification accuracies for spectra derived from three intracortical recordings in S1 from GAERS were significantly higher (balanced accuracy GCCC1841 = 78.8%) compared with those in WAG/Rij rats (balanced accuracy GCCC1841 = 78.8% vs balanced accuracy CCC = 71.5%, *p* < 0.05). Of note, this strain difference could not be attributed to the difference in the amount of training samples (i.e., 1841 derived from 70% of the six 24-h recordings of GAERS vs 161 derived from 70% of the six 4-h recordings of WAG/Rij rats), as a reduction of the training data in GAERS still resulted in higher classification accuracies than in WAG/Rij rats (balanced GCCC161 = 73.6% vs balanced accuracy CCC = 71.5%, *p* < 0.05; Fig. 4*B*).

In order to evaluate whether classification accuracy of the random forest depends on the level of sensitivity achieved by the Maksimenko algorithm, classification performance in GAERS and WAG/Rij rats achieved at sensitivities of 60% and 90% were compared for spectra derived in recordings of Layers IV, V, and VI in S1. In both strains, a small but significant reduction in classification accuracy was noted for spectra derived at a 90% sensitivity as compared with spectra derived at a 60% sensitivity (balanced accuracy CCC = 71.5% vs CCC90% = 63.3% *p* < 0.001; GCCC1841 = 78.8% vs GCCC90% = 73.1% *p* < 0.001). Of note, classification accuracies for spectra derived at a sensitivity of 90% significantly exceeded chance level classification as indicated by surrogate statistics (both *p* < 0.01; Fig. 4*B*). Moreover, accuracies gradually increased toward a maximum at ∼16 trees (Fig. 4*E*).

For out-of-sample evaluation, the random forest trained on spectra derived from three intracortical recordings in S1 of GAERS at a sensitivity of 90% was confronted to spectra derived from 24-h recordings in a separate group of GAERS (*n* = 9).

Table 2 depicts the achieved balanced accuracies of each rat as well as the average confusion matrix, specifying the relative percentage of true positives that had been classified as such (lower right corner), true positives that had been incorrectly classified as false positives (lower left corner), false positives correctly classified as such (upper left corner), and false positives incorrectly classified as true positives (upper right corner). Classification performance drastically dropped and above chance classification tested by permutation statistics was only achieved in a single rat (i.e., rat 5, balanced accuracy 59.62%, *p* < 0.05).

**Table 2:** Out-of-sample performance of the random forest (trained in an undersampling approach on spectra derived from three intracortical recordings in S1 of GAERS at a sensitivity of 90%) confronted to spectra derived from 24-h recordings in a separate group of GAERS (*n* = 9)

	Average confusion matrix	
	Predicted as false positive	Predicted as true positive
False positive	52.46 ± 9.38%	47.54 ± 9.38%
True positive	50.66 ± 8.95%	49.34 ± 8.95%
	Balanced accuracy	F1 score
Rat 1	47.37%	14.53%
Rat 2	53.68%	6.89%
Rat 3	47.44%	11.74%
Rat 4	49.07%	4.82%
Rat 5	59.62% *	9.14%
Rat 6	51.06%	5.44%
Rat 7	51.93%	7.18%
Rat 8	50.13%	4.25%
Rat 9	47.82%	9.68%

Depicted in the upper panel is the average confusion matrix (±SEM), specifying the percentage of true positives correctly classified as true positives (lower right), true positives incorrectly classified as false positives (lower left), false positives correctly classified as false positives (upper left), and false positives incorrectly classified as true positives (upper right). Lower panel depicts the balanced accuracies and F1 scores for each individual rat. Note that the F1 score reflects the trade-off between false alarm rate/sensitivity. Low F1 scores are reflecting the drop of sensitivity associated to the drop of false alarm rate. As our goal in this work is the latter, the low scores are justified by the high balanced accuracies; * denotes an above chance balanced accuracy of classification as verified by surrogate statistics.

As the low performance of the random forest in the out-of-sample evaluation might be attributed to random undersampling (i.e., the algorithm was trained with a training set which does not adequately represent the full spectrum/variance of the false positive spectra), we next evaluated the performance of an random forest, which was trained in a (moderate) oversampling approach. In this approach, the random forest was trained with four times all true positive detections and a matched number of randomly selected false positive detections, derived in three intracortical recordings in S1 of GAERS at a sensitivity of 90% (for details, see Materials and Methods). Again, for out-of-sample evaluation, the trained random forest was confronted to spectra derived from 24-h recordings in a separate group of nine GAERS. Table 3 depicts the achieved balanced accuracies of each individual rat as well as the average confusion matrix.

**Table 3:** Out-of-sample performance of the random forest (trained in an oversampling approach on spectra derived from three intracortical recordings in S1 of GAERS at a sensitivity of 90%) confronted to spectra derived from 24-h recordings in a separate group of GAERS (*n* = 9)

	Average confusion matrix	
	Predicted as false positive	Predicted as true positive
False positive	71.38 ± 2.56%	28.62% ± 2.56%
True positive	46.00 ± 4.00%	54.00 ± 4.00%
	Balanced accuracy	F1 score
Rat 1	70.28%*	46.88%
Rat 2	55.14%	7.59%
Rat 3	60.13%*	16.60%
Rat 4	63.98%*	12.21%
Rat 5	63.15%*	12.02%
Rat 6	59.70%*	8.64%
Rat 7	68.47%*	13.14%
Rat 8	59.00%*	6.51%
Rat 9	64.38%*	19.71%

Depicted in the upper panel is the average confusion matrix (±SEM), specifying the percentage of true positives correctly classified as true positives (lower right), true positives incorrectly classified as false positives (lower left), false positives correctly classified as false positives (upper left), and false positives incorrectly classified as true positives (upper right). Lower panel depicts the balanced accuracies and F1 scores for each individual rat. Note that the F1 score reflects the trade-off between false alarm rate/sensitivity. Low F1 scores are reflecting the drop of sensitivity associated to the drop of false alarm rate. As our goal in this work is the latter, the low scores are justified by the high balanced accuracies; * denotes an above chance balanced accuracy of classification as verified by surrogate statistics.

Taking this (moderate) oversampling approach, the achieved balanced accuracies of the random forest significantly increased (*F*_(1,8)_ = 26.8, *p* < 0.001, *R*^2^ = 0.7), and above chance classification could be achieved in all subjects except one (permutation statistics, all but one *p* < 0.05; Table 3).

Classification of the random forest trained with the (moderate) oversampling approach resulted in a strong reduction in the false alarm rate. While the Maksimenko et al.,(2017) algorithm alone produced an average number of 9388 false alarms within the 24 h, sorting of the random forest reduced the false alarm rate by 71.4 ± 2.6%. Reduction of the false alarm rate, however, occurred at some trade-off between false alarm rate and sensitivity. Here, Maksimenko et al.,(2017) on average correctly predicted 368 out of 409 SWDs, while 40 SWDs were not detected (corresponding to a sensitivity of 90%). Following sorting by the random forest, an average of 200 out of 409 SWDs were correctly predicted (corresponding to a sensitivity of 49%). It has to be mentioned, however, that rather large interindividual differences occurred in prediction performance using the combined “Maksimenko et al. + random forest” algorithm. Highest performance was seen in a rat in which 349 out of 520 SWD were correctly predicted (corresponding to a sensitivity of 67%).

## Discussion

The current study was designed to improve the prediction of SWDs, a type of generalized seizures seen in several forms of absence epilepsy (Panayiotopoulos et al., 1992). While these types of seizures have long been regarded as stochastic events (Lopes Da Silva et al., 2003), a recent study by Maksimenko et al. (2017) aimed at prediction of SWDs through the use of a dedicated algorithm, which calculates the product of the wavelet energy in LFP recordings taken at three locations in the cortico-thalamic system of absence epileptic rats. A drawback was that this algorithm suffered from a large amount of false positive detections. Therefore, the current study was designed to improve prediction performance, as quantified by sensitivity, specificity and balanced accuracy of prediction. The rational was to systematically vary the sites of simultaneous recordings in the cortico-thalamic system, including somatosensory and motor cortices, rostral and caudal RTN, specific (VPM) and higher order thalamic nuclei (Po, ATN), in view of their distinct role in initiation, spread and synchronization of SWDs (Lüttjohann and van Luijtelaar, 2015; Depaulis et al., 2016; Crunelli et al., 2020). Results were iteratively analyzed, in that all possible combinations of the 2–3 simultaneous recording sites were compared by using the algorithm of Maksimenko et al. (2017). Moreover, a thorough comparison of wavelet spectra corresponding to true and false positive detections was performed, and a random forest machine learning algorithm was trained to further differentiate between true and false positives. Algorithm performance was evaluated according to the guidelines of good scientific practice (Mormann et al., 2007; Kuhlmann et al., 2018; long-lasting, non-selected, pseudo-prospective 24-h recordings with both in-sample and out-of-sample periods, evaluation against chance level prediction using surrogate statistics), and it was found to reduce the false alarm rate by on average 71.4%

### Highest SWD prediction performance is achieved with analysis of LFP signals in the close proximity of the seizure initiation network in S1

Comparison of a total of 85 combinations of recording sites within the cortico-thalamic system (Table 1), revealed that prediction performance was best when based on analysis of the wavelet energy of recordings obtained by three recording electrodes within the deep layers of the S1. SWDs are well known to be generated in the cortico-thalamic system. While the exact interactions between cortex and thalamus are still a matter of debate, accumulating evidence indicates that SWDs originate from a local intracortical initiation network in the peri-oral region of the S1 (Meeren et al., 2002; Jarre et al., 2017; Crunelli et al., 2020). In GAERS, the crucial role of Layers V and VI of S1 has been highlighted, as theses layers were found to contain abnormally (i.e., hyperactively) discharging neurons, which drove neuronal activity in other cortical layers as well as thalamic activity (Polack et al., 2007; Lüttjohann and Pape, 2019). These epileptogenic neurons display activity patterns strikingly similar to the precursor oscillations detected by the algorithm in the present study, including an increase in activity within up to two seconds before SWD onset and a firing frequency of ∼10 Hz (Polack et al., 2007). Highest sensitivity of prediction was achieved by the Maksimenko et al. (2017) algorithm based on analysis of wavelet energy in the deep layers of S1 (IV, V, VI), which significantly outreached all other cortico-thalamic and intrathalamic combinations of recording sites (Fig. 2*A*). Moreover, further classification of true and false positive detections by a trained random forest also reached highest, above chance balanced accuracies for spectra derived in the deep layers of S1, while classification based on intrathalamic-spectra failed to achieve above chance balanced accuracies (Fig. 4*B*). These data are in line with the concept of a local intracortical initiation network in S1 (Meeren et al., 2002; Polack et al., 2007).

Interestingly, prediction performance of the Maksimenko et al. (2017) algorithm significantly dropped on reducing the number of simultaneous recordings sites in the deep somatosensory layers from three to two (Fig. 2), further demonstrating the importance of local intracortical synchronization in S1 for SWD generation. The concurrent increase in the false alarm rate might indicate a lack of information concerning the generation of other synchronized oscillations, which might be transmitted to the deep cortical layers by other subcortical structures (Depauls et al., 1990; Sitnikova et al., 2009).

The sensitivity of SWD prediction based on three simultaneous recordings in S1 also outreached sensitivity achieved in deep layers of M2. In view of long-range intracortical connections between S1 and M2, specifically from Layer V/VI of S1 to Layer V of M2 (Condé et al., 1995; Zhang and Deschênes, 1997; Reep and Corwin, 1999; Zakiewicz et al., 2014), the high SWD prediction performance in S1 compared with M2 suggests that SWD precursor activity is a locally restricted cortical phenomenon, at least with regard to the initiation zone in S1.

Prediction performance of the Maksimenko et al. (2017) algorithm was found to differ between the two genetic model strains, in that prediction performance was generally more accurate and spectra corresponding to true and false positive detections were more differentiated in GAERS compared with WAG/Rij rats. Differences between the two models and even between different colonies of the same strain have been described for distinct electrographic features of the SWDs (Akman et al., 2010; Powell et al., 2014). It is likely that that the frequency band W_(5–10 Hz)_, employed by the algorithm for precursor detection, better suits detection of 5–9 Hz oscillations, which have been described to preceded SWDs in GAERS (Pinault et al., 2001). In WAG/Rij rats, on the other hand, precursor activity has been described in both theta and δ frequency bands (van Luijtelaar et al., 2011, 2016), implying that improved SWD prediction performance in WAG/Rij rats might require additional fine-tuning of the frequency band width applied by the Maksimenko et al. (2017) algorithm.

### Random forest machine learning algorithm for the reduction of false alarms

Irrespective of the combination of recording sites, false alarm rates remained at a relatively high level. However, statistical comparison between wavelet spectra of true positive and false positive predictions revealed significantly different wavelet energies in both strains. Furthermore, a random forest machine learning algorithm could be trained to detect such preexisting spectral differences to further differentiate between true and false positive predictions. In long-lasting, out-of-sample, 24-h recordings in the deep layers of S1 in nine GAERS, which cover the full diurnal variation reported for SWD occurrence (Smyk and van Luijtelaar, 2020), this additional classification of a trained random forest reduced the false alarm rate for SWD prediction by an average of 71.4%

Of note, the balanced accuracy of classification depended on the approach of training (i.e., oversampling vs undersampling) introduced to the random forest. Machine learning algorithms require a balanced training set in order for unbiased assessments of error rates to be achieved (Khan et al., 2018). With respect to SWDs, precursor and true positive predictions are an underrepresented class compared with the much larger group of interictal and false positive predictions. For balance training, random undersampling and (moderate) random oversampling (Kubat et al., 1997; Chawla et al., 2002) were used, and classification performance of two differentially trained random forests were compared. Significantly higher balanced accuracies were found for the random forest trained in the moderate oversampling approach as compared with the under-sampling approach, suggesting that undersampling does not include the full spectrum of variance among different types of false positive detections.

Another common source of error in machine learning algorithms is the choice of the dataset on which algorithm performance is evaluated. In line with guidelines of good scientific practice (Mormann et al., 2007; Kuhlmann et al., 2018), algorithm performance was evaluated both in unseen in-sample recordings of the same rats (30% of unseen data) as well as in lasting, non-selected, pseudo-prospective 24-h recordings acquired in a separate group of GAERS (out-of-sample evaluation). The importance of such an additional validation step can readily be inferred from the drop in algorithm performance between in-sample and out-of-sample testing. Furthermore, our attempt to confront the algorithm with the full range of diurnal variations necessitated these 24-h recordings.

Unfortunately, classification by the random forest also went along, to some degree, with a decrease in prediction sensitivity, in that 200 out of 409 SWD were correctly predicted (corresponding to a decrease in sensitivity by 41%). The prediction of SWDs thus lacks behind the performance of prediction systems aimed at focal convulsive seizures, reaching sensitivities of prediction up to ∼90% (Kiral-Kornek et al., 2018; Khan et al., 2018; Kuhlmann et al., 2018). Of note, SWDs in absence epilepsy constitute a type of seizure that is fundamental different from focal convulsive seizures, in terms of pharmacological profile, frequency of occurrence, pathomechansms, and interictal spike patterns (Depaulis and van Luijtelaar, 2006). Moreover, the moderate performance of SWD prediction may relate to interindividual differences, which are visible in both in-sample and out-of-sample validation. Spatial variance between the position of the recording electrodes relative to the initiation zone in S1, or neurobiological differences in the cortical initiation network between individuals (Meeren et al., 2002) may explain these findings. As a corollary, individualized training of the random forest on long-term data obtained from a single individual may fine-tune and improve random forest approaches to SWD prediction.

### Possible translation to prediction of SWDs in human absence epilepsy

SWD prediction performance of the Maksimenko et al. (2017) algorithm and combined classification performance of the random forest was best for intracortical recordings obtained in close proximity to the seizure initiation network in S1. These findings provide an interesting perspective for SWD prediction in humans using surface EEG recordings. As in the genetic rat models, a local cortical initiation site of SWDs has been identified using EEG and MEG recordings combined with nonlinear association analysis in children with absence epilepsy (Westmijse et al., 2009; Ossenblok et al., 2019). Moreover, Gupta et al. (2011) identified preictal sources of activity, occurring ∼1 s before SWDs. Of note, the exact location of the cortical SWD onset zone is variable between individual children and preictal activity was reported to be most pronounced in the δ frequency range. Fine tuning of the frequency bands analyzed by the Maksimenko et al. algorithm, and training of the random forest on long-lasting EEG recordings in an individual child, are thus promising possibilities paving the way for SWD prediction in children.

Wavelet analysis is a fast and reliable method for assessing non-stationary signals like LFP or EEG recordings (Hramov et al., 2015). Together with the fast temporal precision of EEG and LFP recordings, this approach allows a detection of fast and short-lasting events like SWD precursors and opens the door for an implementation in an on-line setting aimed at real time prediction and prevention (Maksimenko et al., 2017) with as little interference to the overall brain activity as possible (Osterhagen et al., 2010; van Luijtelaar et al., 2017). Such a treatment approach might go along with a strong relief of side-effects often reported for the commonly used chronic pharmaceutical interventions (Crunelli et al., 2020).

## Synthesis

Reviewing Editor: Liset Menendez de la Prida, Instituto Cajal CSIC Decisions are customarily a result of the Reviewing Editor and the peer reviewers coming together and discussing their recommendations until a consensus is reached. When revisions are invited, a fact-based synthesis statement explaining their decision and outlining what is needed to prepare a revision will be listed below. The following reviewer(s) agreed to reveal their identity: NONE.

Your manuscript was evaluated by two independent experts and myself. We agree there is value in your approach to evaluate prediction of electrographic idiopathic absence seizures using multi-laminar spectral information operating on a random forest classifier. However, we have several concerns regarding the depth of the analysis provided, the need for additional contrast and experiments and many other details requiring extensive revision.

While we feel addressing all this may take more than two months (the limit to reject a paper with major revision in eNeuro), we are happy to give you a chance to revise. However, it is important to stress that we expect a thorough revision and that you address all our comments satisfactorily. Please, note that the second review round is definitive for the ms to have real chances in the journal.

Major comments:

1- We appreciate this new work extends a previous method (Maksimenko et al., 2017) for prediction of absence seizures. The new method is able to reduce the number of false positives and better exploit inter-laminar LFP spectral dynamics. However, we find the analysis performed very poor. Most of the manuscript is focused in Maksimenko’s (only one out of six figures are dedicated to results from the random forest) and there is poor comparison with other methods. We feel addressing this requires a full reorganization of the ms. In particular:

a) We miss the most important parts of what any machine learning technique should have: a scheme to conceptualize the method and a parametric search (at least number of features and number of trees).

b) Most decisions regarding training (60% vs 90% of sensitivity, under and oversampling rates, random forest parameters,...) are not justified. At some point the dataset (or a subset) should be evaluated under a single trained random forest to see how generalizable the model is.

c) Similarly, we don’t understand the basis for other decisions on undersampling (why is that neccesary if an equal number of randomly selected false positive detections were taken?) and oversampling (in which four ‘??’ times all true positive detections of randomly selected false positive detections were taken). A more thorough study of all possible combinations of sampling and sensitivities should be shown to justify those numbers.

d) Thresholds are mentioned several times, but we feel an analysis of their implication or effect is lacking. It should be clear how can they affect the result, as threshold are an essential part of any detection algorithm.

e) Why “14 fixed threshold values ranging from 0.1 to 0.75 for all combinations of three recording sites, and a total of 16 fixed threshold values ranging from 0.005 to 0.04 for all combinations composed of two recording sites” was used? You need to support your choise And why is there such a huge difference (one order of magnitude) between ranges for 2 and 3 recording sites? It has to be justified.

f) There is not a thorough comparison of performance and accuracy between this and Maksimenko’s algorithms (except a brief comment in the text) nor with any other existing method or solution.

2- In the same line, representation and analysis of the random forest performance should be improved. The large tables II and III are very difficult to conceptualize, and almost impossible to infer any global behavior. Also, confusion matrices should be in percentage, not in absolute numbers. If that were the case, information could be grouped and presented as four subplots, or as a heat-map with the mean and standard deviation; anything that helps the reader to really grasp information from the data.

3- We also have concerns regarding novelty and optimization of your results. To our eyes, figure 1A is almost the same as figure 1A of Maksimenko et al. (2017). We recommend to improve Fig.1 in line with our point 2. In addition, we suggest to consider merging figures 1, 2 and 3 (e.g. representation of figure 1B, 2A and 3 looks unnecessary, it can be grouped as in 1C or 2B). Figures 3 and 4 can also be merged. All these changes would make space for the random forest, which should be the main point of the manuscript, as suggested in our previous points).

4- Prediction sensitivity based on recordings in layers 4, 5, and 6 of the primary somatosensory cortex was significantly better than all other combinations of recordings. Thus, this study further supports the cortical focus theory for absence seizures and provide a proof of concept for the possibility to predict absence seizures from human patients. However, a real advance in the temporal extent of seizure prediction [∼1s] compared to previous descriptions of preictal events in thalamo-cortical circuits remains unclear. Preictal changes in the ECoG of GAERS have been described before. For instance Polack et al. (2007) showed they are characterized by a barrage of oscillations (lasting up to 1 s) with a slightly higher internal frequency (∼ 10 Hz) than full-blown SWDs. These seizure precursors also occur concomitantly in the underlying pyramidal neurons, specifically in layer 5-6 of the primary somatosensory cortex (Depaulis and Charpier, 2018). These preictal oscillations are very similar to those described in this article, as illustrated in Figure A1. We would like to better understand to what extent do the present results improve the prediction of seizures as can be done on the basis of these preictal oscillations? What is the temporal extent of the current prediction and how does it compare to those neurophysiological prodromes? This can be addressed both quantitatively (as in point 1f) and qualitatively (at the Introduction and discussion).

5- It is mentioned that in some WAG/Rij rats, LFPs were also captured from the “secondary motor cortex”. What is the rationale for this choice? What are the anatomical connections between this cortical area and the seizure-initiating cortex, i.e. S1? Also, no recordings were made in layers II-III of S1 (or M2). This may be a serious shortcoming in the present study, because pyramidal neurons in the upper layers are an important neural relay in the flow of thalamo-cortical information to the deep layers, where SWD first occurs. We would like you provide recordings from the upper layer to support/tune your conclusions.

6- The criteria used to determine the onset of SWD are unclear. In Figure 1A, the indicated onset of SWD (channel 1) is actually preceded by relatively large and rhythmic deflections that could be consistent with epileptic activity. The account: “Onset of SWDs was earlier determined by a trained electrophysiologist.” may reflect biases regarding divergences between experts.

Other comments:

7-What are the properties of the recording electrodes used in the Münster laboratory? How was the distinction between the different layers made? It is mentioned that a histological confirmation of the recording sites was done. Can the authors provide an example of histological distinction between the different cortical layers? How many electrodes were inserted together? This is not clear because it is mentioned that 85 combinations were examined from a relatively small set of experiments.

8-In the first part of the results, the site of recording of the LFPs is not indicated (which cortices, which layers?) and there is no reference to an appropriate figure. However, it is stated that “All differences in LFP morphology between strains and recording sites are consistent with previously published data.” What are these differences? It would be helpful to provide typical examples of LFPs recorded in the different regions tested, as in Figure 1A (where the recording sites are not indicated). This also raises the question of the possible impact of spike wave morphologies on detection accuracy and prediction performance

9-GAERS is first mentioned in the introduction without any reference and brief description of the model.

10-We found section 2.2 very difficult to follow if section 3 hasn’t been read. As section order is imposed by the journal guideliness, we recommend to rewrite the narrative in order to improve readibility

11- A total of 24 WAG/Rij rats are mentioned in line 72, but only 16 are mentionned in line 77 Please, clarify.

12- You specifically mention that eight different brain structures are recorded for WAG/Rij rats. How many are recorded for GAERS rats? We are not clear if this may be the reason why combinations of regions (Table I) are done, but only for two and three regions. PLease clarify also if this is the reason for (line 287) “predictions based on three recording sites reached significantly higher sensitivities” and make this clear for GAERS ad WAG/Rij animals.

13- The number of possible combinations of 3 elements out of 8 without repetition are 56, but in line 123, 57 are stated. Please, clarify

14- Section 2.2.1 refers to many wavelet-related concepts. We are unclear whether they are different or not: “wavelength energy” (line 132), “wavelet energy” (line 134), “wavelet spectra” (line 183), “wavelet power” (line 190), “wavelet features” (line 213). Please, clarify and try to be consistent.

15- As S1 is a brain region, ΔS1 could be confusing in line 135.

16- Line 165 should mention figure 2, if line 142 mention figure 1.

17- Line 184, why 50 randomly selected FP detections? Please, justify.

18- Line 333, extra comma.

19- Line 377, average balance accuracy is mentioned for the first time, but described later (see line 434).

20- Refering to “(slight)” seems as if authors weren’t sure if it is good or bad using an oversampling approach.

21- X and Y labels on figure 5 B and C are missing. Also label of the heat-map legend.

22- Y label on figure 5 E

23- We recommend to use consistent representations for performance-related data (figures 1B, 1C, 2A, 2B, 3, 6A). Please, consider reporting data according to estimation statistics guidelines: either boxplot, mean and standard deviations plus individual data points, etc... Please use the same standard along the paper.

24- The discussion is too long and focuses on methodological issues. The central scientific question and the main result of the study (the prediction of epileptic activity) are drowned out by sophistical technical considerations. The discussion would benefit from being more balanced on the new data obtained.
