# Supplementary material for: Seizure Prediction in Genetic Rat Models of Absence Epilepsy: Improved Performance through Multiple-Site Cortico-Thalamic Recordings Combined with Machine Learning
Source: eNeuro. 2022 Feb 8;9(1):ENEURO.0160-21.2021. doi: 10.1523/ENEURO.0160-21.2021 (PMC8856717; doi:10.1523/ENEURO.0160-21.2021)
Supplement: Extended Data 1 — RF oversampling seizure prediction. Download Extended Data 1, ZIP file. [file enu-eN-NWR-0160-21-s01.zip › RF_oversampling_seizure_prediction.pdf]

```python
import pandas as pd
import os
import numpy as np
from glob import glob
import keras
from keras import backend as k
from keras.metrics import accuracy, categorical_accuracy
from sklearn.model_selection import KFold, cross_val_predict, train_test_split
from sklearn.metrics import log_loss, confusion_matrix, f1_score, classification_report, precision_score, balanced_accuracy_score
from sklearn.ensemble import RandomForestClassifier
from datetime import datetime
from imblearn.over_sampling import RandomOverSampler


def load_filenames():
    """
    Load all extracted files from local storage.
    Files are in the form of *.dat. A collection of all filepaths will be created in the variable filename.
    Data location is in variable fileordner and path_over (i.e. -> C:\\Users\\Main\\Desktop\\Data2\\)
    """
    path_over = "Data2\\"
    os.chdir(fileordner+path_over)
    filename = [glob(e) for e in ['*.dat']]
    filename = filename[0]
    return filename


def load_files():
    """
    Preselection of channels within all files. Variables X and Y are loaded.
    filename is imported from the function load_filenames and varibale for X and Y are created (def_x and def_y).
    All files will be looped over and X and Y will be corretly added to the variables def_pre_x and def_pre_y.
    Here in column "0" the boolean value is provided and then sorted to def_pre_y.
    Columns 2-10 will be sorted to the df_pre_x as here are the information stored.
    Finally, all the pre data variables will be concatenated into a singular variable (def_x and def_y) for later use.
    """
    filename = load_filenames()
    df_x = []
    df_y = []
    for i in filename:
        df_pre_x = pd.read_csv(i, header=None, index_col=None, sep='   ', usecols=[2, 3, 4, 5, 6, 7, 8, 9, 10], engine='python')
        df_pre_y = pd.read_csv(i, header=None, index_col=None, sep='   ', usecols=[0], engine='python', dtype=np.int8)
        df_x.append(df_pre_x)
        df_y.append(df_pre_y)
    df_x = pd.concat(df_x)
    df_y = pd.concat(df_y)
    return df_x, df_y


def data_preparation_part(ratio):
    """
    Preparation of data.
    df_x and df_y are loaded into this function and the positions of true and false predictions will be defined.
    An array will be created from the positions of the true and false predictions.
    Ratio between true and false class will be defined by variable "ratio".
    As not all data will be used to train the random forest, a preselection is done from the sample_pick variable.
    This selection will then be exported out of the function via the variables x_train
```

```python
    and y_train.
    """
    global ratio
    df_x, df_y = load_files()
    # Class 1 = false positives // Class 2 = true positive(precursor)
    position_class_1, position_class_1_null = np.where(df_y == 0)
    position_class_2, position_class_2_null = np.where(df_y)
    # pick a specific ratio (a) between each class
    np.random.seed(100)
    sample_pick = np.random.choice(position_class_1, len(position_class_2)*ratio)
    sample_pick = np.concatenate((sample_pick, position_class_2), axis=0)
    # mini extraction of always the same test data
    y_train = np.array(df_y)[sample_pick]
    x_train = np.array(df_x)[sample_pick]
    return x_train, y_train


def balanced_accuracy(y_true, y_pred):
    """ Custom metric based on sensitivity and specificity"""
    true_positives_sensit = k.sum(k.round(k.clip(y_true * y_pred, 0, 1)))
    possible_positives_sensit = k.sum(k.round(k.clip(y_true, 0, 1)))
    true_negatives_specificity = k.sum(k.round(k.clip((1 - y_true) * (1 - y_pred), 0, 1)))
    possible_negatives_specificity = k.sum(k.round(k.clip(1 - y_true, 0, 1)))
    return (((true_positives_sensit / (possible_positives_sensit + k.epsilon())) + (true_negatives_specificity / (possible_negatives_specificity + k.epsilon())))/2)


def rand_forest(Trees, seed, ratio):
    '''
    Random forest training.
    x_train and y_train will be imported from the cuntion data_preparation_part with the ratio.
    This array will be split into a test and training part with a ratio of 30% testing and 70% training.
    The random forest will be trained with a balanced class_weigt and a modifiable amount of trees by the variable Tree_count.
    The output of this function will be balanced_accuracy and a confusion matrix.
    '''
    x_train, y_train = data_preparation_part(ratio)
    np.random.seed(seed)
    x_train, x_test, y_train, y_test = train_test_split(x_train, y_train, test_size=0.3)
    ros = RandomOverSampler()
    x_train, y_train = ros.fit_resample(x_train, y_train)
    x_test, y_test = ros.fit_resample(x_test, y_test)
    Tree_Count = Trees
    y_train = np.eye(2)[y_train]
    y_test = np.eye(2)[y_test]
    clasifier = RandomForestClassifier(n_estimators=Tree_Count, class_weight='balanced')
    clasifier.fit(x_train, y_train)
    pre = clasifier.predict(x_test)
    conf_matrix = confusion_matrix(y_test.argmax(axis=1), pre.argmax(axis=1))
    # Output
    a = (balanced_accuracy_score(y_test.argmax(axis=1), pre.argmax(axis=1)))
    cm = np.array2string(conf_matrix)
    print(a)
    print(cm)


def itter(Trees, seed, ratio):
    rand_forest(Trees, seed, ratio)


if __name__ == '__main__':
    '''
    Location of data in variable fileordner
    '''
```

```python
fileordner = 'C:\\Users\\bjoer\\Desktop\\'
'''
The following command will loop over several seeds (0-100) with different ratios. The random forest is defined with 1000 Trees.
'''
for i in range(100):
    print('Shuffle random Nr: ',i)
    #print('Oversampling 1:1')
    itter(1000, i, 1)
    #print('Oversampling 2:1')
    itter(1000, i, 2)
    #print('Oversampling 3:1')
    itter(1000, i, 3)
    #print('Oversampling 4:1')
    itter(1000, i, 4)
    #print('Oversampling 5:1')
    itter(1000, i, 5)
    #print('Oversampling 6:1')
    itter(1000, i, 6)
    #print('Oversampling 7:1')
    itter(1000, i, 7)

'''
The following command will loop over several seeds (0-100) with a ratio of 1:1. The random forest is defined with 1000 Trees.
'''
for i in range(0,100):
    itter(1000, i, 1)
```
